# Respiratory health and chronic disease risks in residents of agricultural areas in Chiang Mai, Northern Thailand

**DOI:** 10.1371/journal.pone.0321471

**Published:** 2025-04-04

**Authors:** Anurak Wongta, Supansa Pata, Kriangkrai Chawansuntati, Supachai Yodkeeree, Surat Hongsibsong, Woottichai Khamduang

**Affiliations:** 1 School of Health Science Research, Research Institute for Health Sciences, Chiang Mai University, Chiang Mai, Thailand; 2 Research Institute for Health Sciences, Chiang Mai University, Chiang Mai, Thailand; 3 Department of Medical Technology, Faculty of Associate Medical Science, Chiang Mai University, Chiang Mai, Thailand; 4 Department of Biochemistry, Faculty of Medicine, Chiang Mai University, Chiang Mai, Thailand; Christian Medical College, INDIA

## Abstract

Respiratory health and chronic disease risks are prevalent concerns in agricultural communities in Northern Thailand, prompting an assessment of these issues among residents in Chiang Mai. A cross-sectional study involving 145 participants was conducted in San Pa Tong District from October to December 2023, utilizing structured questionnaires and spirometry tests to evaluate lung function and chronic health disease risk. The study found that education and income significantly impacted lung function, with better FVC% and FEV1/FVC% predicted values observed in those with primary education and lower income. Lower DM risk was associated with better lung function (P =  0.023). Logistic regression showed a significant increase in lung function impairment among participants with high to very high DM risk (aOR 9.06, p < 0.05). High CV and COPD risk levels also correlated with lung function impairment, though not all results were statistically significant. The results emphasize the intricate interplay between socioeconomic factors, chronic disease risks, and lung function, advocating for public health interventions that holistically address population respiratory and metabolic health.

## Introduction

Agriculture in Northern Thailand plays a crucial role in the economy by supporting various crops and livestock farming activities [1]. These activities, however, pose significant health hazards, including respiratory complications and persistent ailments such as diabetes mellitus (DM), cardiovascular disease (CVD), and chronic obstructive pulmonary disease (COPD). The burning of agricultural residue releases air pollutants that can cause acute cardiopulmonary illnesses, necessitating the implementation of effective policies to address this issue [2,3]. Previous research has shown that poor working conditions affect farmers’ quality of life, highlighting the need for improvements in occupational safety [4]. A combination of climate data, socioeconomic factors, and crop suitability assessments is crucial for developing strategies to minimize the effects of climate variability on crop yield and farm profits [1].

Respiratory health issues are a significant concern in agricultural communities owing to various occupational hazards, including exposure to pesticides, organic dust, animal dander, and other airborne particulates [5,6]. Seasonal variations in air quality, particularly high levels of particulate matter (PM) 2.5, exacerbate respiratory conditions on farms and ranch operators. Research has linked agricultural work to respiratory ailments, highlighting the need for further research on health outcomes in this population [6–8]. Effective preventive measures and respiratory protection strategies are essential for safeguarding respiratory health.

Chronic diseases such as DM, CVD, and COPD significantly impact an individual’s quality of life and overall health due to a complex interplay of genetic, environmental, and lifestyle factors [9,10]. In agricultural communities, the risk of developing these chronic conditions is heightened due to occupational exposure and intertwined lifestyle choices [11]. Studying disease rates and risks in farming is important for creating effective public health strategies and improving health outcomes. Addressing specific challenges in agricultural environments enables the development of targeted strategies to enhance the community health and well-being [12].

Chronic exposure to PM2.5 is linked to declining lung function and exacerbation of noncommunicable diseases (NCDs), such as COPD. PM2.5, composed of elements like arsenic, nickel, and vanadium, contributes to DNA methylation and inflammation, thereby reducing lung function parameters such as forced expiratory volume in the first second (FEV1) and forced vital capacity (FVC) [13]. In COPD patients, PM2.5 exacerbates lung inflammation and fibrosis, increasing inflammatory cytokines and collagen deposition [14]. Long-term exposure to PM2.5, and its components, such as black carbon and nitrate, are associated with more outpatient visits and hospitalizations, indicating a wider public health impact [15]. While physical activity can benefit COPD patients, it also raises exposure to PM2.5 and ozone (O3). However, physical activity may mitigate the adverse effects of nitrogen dioxide (NO2) on lung function [16].

Despite these recognized issues, comprehensive data on respiratory health and chronic disease risks in Chiang Mai’s agricultural areas are limited. This cross-sectional study aims to assess respiratory function and chronic disease risk scores, and their correlation with spirometry results. The findings are intended to guide public health policies and interventions, identifying high-risk groups for health promotion and disease prevention efforts.

## Materials and methods

### Apparatus

Spirometry examinations were conducted utilizing a Spirometer device (SpiroScout, Ganshorn, UK).

The VITROS XT7600 analyzer (CardinalHealth, USA) was employed for the analysis of blood glucose and cholesterol concentrations.

### Target participants

This cross-sectional study was conducted between October 30 and December 30, 2023, in the San Pa Tong District of Chiang Mai, Thailand. The G * Power 3.1 software was utilized to calculate the number of participants. To achieve a study power of 95% with a Type I error rate of 0.05, and an effect size of 0.3, indicating a required sample size of 134 individuals. We enrolled 145 participants to account for potential spirometry test failures and incomplete responses. Participants aged 18 and above were recruited through convenience sampling based on inclusion criteria and willingness to participate. Data was collected through structured questionnaires administered during face-to-face interviews. The survey gathered comprehensive information on demographic characteristics, DM risk scores, CV risk scores, COPD risk scores, and spirometry results.

### Ethics approval and consent to participate

This study was approved by the ethics review board before data collection (Doc No. AMSEC-66EX-062) from the Faculty of Associate Medical Technology, Chiang Mai University. Consent was obtained from all participants after receiving comprehensive information regarding the study, and their agreement was documented in written form.

### Data collection

#### Questionnaires survey.

Trained research assistants conducted face-to-face interviews using structured questionnaires, which were systematically organized into four key sections to gather a comprehensive range of data:

1. Demographics: This foundational section collected essential background information about the participants, including age, sex, educational level, and farming experience.
2. DM Risk Scores: The risk of diabetes was assessed using the Thai Diabetes Risk Score, which considers factors such as age, body mass index (BMI), waist circumference, blood pressure, glucose level, and family history of diabetes. This score helps identify individuals at high risk of developing diabetes [17].
3. CV Risk Scores: The risk of cardiovascular diseases was measured using the Thai Cardiovascular Risk Score. This score includes variables such as age, sex, smoking status, blood pressure, and cholesterol levels. This aids in evaluating the likelihood of future cardiovascular events and guides preventive measures [18].
4. COPD Risk Scores: The risk of COPD was evaluated using the COPD Assessment Test (CAT). This score is instrumental in identifying individuals who may benefit from early intervention to prevent or manage COPD [19].

#### Blood collection.

Venipuncture was performed to collect 10 ml of blood from each participant. Trained medical personnel carried out the blood collection under sterile conditions. The blood samples were then processed and analyzed for glucose and cholesterol levels using a VITROS XT7600 analyzer.

### Spirometry procedure

The FVC maneuver was performed using standardized procedures and calibrated spirometers. Participants were instructed to avoid large meals, vigorous exercise, and smoking for at least two hours before the test. Seated comfortably and using a nose clip, participants received detailed instructions and demonstrations. The procedure involved taking a deep breath to full lung capacity and forcefully exhaling into the spirometer. The FVC, FEV1, and the FEV1/FVC ratio were calculated. Each participant performed the maneuver at least three times, and the highest FVC and FEV1 values were recorded. Acceptable maneuvers were artifact-free, had a rapid start, and featured smooth, continuous exhalation, ensuring the differences between the largest and second-largest FVC and FEV1 values were within 150 ml. Spirometry data were used to classify lung function into normal, and impaired lung function including both restrictive and obstructive patterns to identify respiratory issues.

### Data analysis

Data were analyzed using SPSS v20, and descriptive statistics were used to summarize the participant demographics and chronic health risk group. Spirometry results were analyzed using the Mann-Whitney U Test and Kruskal-Wallis Test based on demographics and health risk levels. Health risk levels and lung function impairment results were examined using logistic regression analysis and found to be significant at p-value (P) <  0.05.

## Result

The demographic characteristics of the 145 participants (78% women and 32% men) were presented in Table 1. The majority were under 61 years of age (52%) followed by those in the older age group (48%). Most had a primary education (58%), while the remaining 42% had education levels higher than primary school. The income distribution ranged from less than 5,000 Bath per month (52%) to more than 5,000 Baht per month (48%). Occupational status included agricultural workers (27%) and nonworkers (73%). The prevalence of chronic health risk showed the most common groups were those with moderate to high risk diabetes (33%), Low risk CV (72%), and low-risk COPD risk group (90%).

**Table 1:** Demographic characteristics of study participants (N = 145).

Demographical characteristic		Frequency n (%)
Sex	Man	32 (22)
	Woman	113 (78)
Age (years)	≤ 60	76 (52)
	> 60	69 (48)
Education levels	Primary school	84 (58)
	Higher than primary school	61 (42)
Income (Baht/Month)	≤5,000	75 (52)
	>5000	70 (48)
Agricultural worker	No	106 (73)
	Yes	39 (27)
DM risk level	Low risk	34 (24)
	Low to moderate risk	29 (20)
	Moderate to high risk	48 (33)
	High risk	16 (11)
	Very high risk	18 (12)
CV risk level	Low risk	109 (72)
	Moderate risk	30 (20)
	High risk	4 (3)
	Very high risk	2 (1)
COPD risk level	Low risk	137 (90)
	High risk	8 (5)

DM, Diabetes mellitus; CV, Cardiovascular, COPD, Chronic obstructive pulmonary disease.

The spirometry findings indicated significant variances associated with demographic traits and chronic disease risk as shown in Table 2. Men had a lower median FVC% predicted (89.90) than women (91.14), though not statistically significant (P =  0.61). Participants aged 60 or younger exhibited a slightly lower FVC% predicted (90.11) than older individuals (92.29), P =  0.774. Education level was associated with FVC% predicted, with primary school graduates showing higher values (93.51) than those with greater education (87.81), P =  0.020. However, as this study is cross-sectional, this association does not imply causation. The relationship may be influenced by underlying contextual and environmental factors such as occupational exposure, socioeconomic status, or healthcare access. Income also significantly affected lung function, as those earning below 5,000 Baht per month had higher FEV1/FVC% predicted ratios (103.09) than their higher-income counterparts (100.34), P =  0.046. No significant lung function differences were noted between agricultural workers and non-workers. However, participants with lower DM risk displayed improved FVC% predicted values (95.61) compared to higher-risk individuals, with P =  0.023 indicating significance. Differences in CV risk levels were not significant, yet those with low COPD risk had a notably higher FEV1/FVC% predicted ratio (102.02) than the high-risk group (93.87), P =  0.043.

**Table 2:** Distribution of spirometry results among participants by demographic characteristics and chronic disease risk groups (N = 145).

Demographical characteristics		Frequency n (%)	Spirometry [median (IQR)]					
FVC% predicted	P	FEV1% predicted	P	FEV1/FVC % predicted	P
Sex	Man	32 (22)	89.90 (81.35-100.04)	0.610	100.37 (82.90-107.81)	0.099	100.69 (95.08-105.58)	0.634
	Woman	113 (78)	91.14 (82.89-96.97)		90.37 (81.42-101.89)		102.03 (94.70-106.90)	
Age (years)	≤ 60	76 (52)	90.11 (82.32-98.02)	0.774	92.47 (83.88-102.50)	0.588	101.00 (95.70-105.62)	0.246
	> 60	69 (48)	92.29 (81.65-99.56)		90.37 (78.47-103.42)		103.09 (92.05-108.07)	
Education levels	Primary school	84 (58)	93.51 (83.06-100.65)	0.020^*^	90.97 (79.00-103.27)	0.707	101.86 (93.04-106.35)	0.422
	Higher than primary school	61 (42)	87.81 (81.74-94.33)		91.71 (84.08-101.93)		101.63 (96.19-107.06)	
Income (Bath/Month)	≤5,000	75 (52)	91.11 (81.91-97.64)	0.464	91.71 (81.37-102.85)	0.828	103.09 (96.02-107.67)	0.046^*^
	>5000	70 (48)	91.38 (82.75-98.76)		91.87 (83.02-103.21)		100.34 (94.42-105.09)	
Agricultural worker	No	106 (73)	90.88 (82.38-96.89)	0.438	92.86 (81.49-102.92)	0.534	102.03 (95.91-107.36)	0.100
	Yes	39 (27)	93.5 (81.48-101.51)		89.69 (81.6-103.18)		99.19 (94.44-105.46)	
DM risk level	Low risk	34 (24)	95.61 (82.70-105.13)	0.023^*^	93.21 (83.95-107.31)	0.099	100.34 (95.00-102.74)	0.195
	Low to moderate	29 (20)	89.08 (83.52-93.89)		89.07 (77.39-99.59)		100.67 (90.47-106.34)	
	Moderate to high	48 (33)	93.51 (83.12-100.54)		98.78 (81.54-104.73)		103.08 (94.26-108.03)	
	High	16 (11)	91.99 (80.32-94.82)		94.10 (79.87-104.14)		104.47 (99.34-107.98)	
	Very high	18 (12)	86.27 (78.03-93.59)		85.98 (74.89-93.43)		102.97 (98.20-107.96)	
CV risk level	Low	109 (72)	91.11 (82.84-98.46)	0.827	92.17 (82.43-102.77)	0.936	101.29 (95.07-106.22)	0.306
	Moderate	30 (20)	91.70 (78.03-98.28)		87.75 (76.43-103.70)		103.76 (95.34-107.95)	
	High	4 (3)	89.79 (79.35-100.41)		93.25 (83.66-107.79)		109.31 (94.76-114.43)	
	Very high	2 (1)	91.61 (89.72-NC)		92.94 (82.70-NC)		95.81 (86.62-NC)	
COPD risk level	Low	137 (90)	91.12 (82.28-97.38)	0.719	92.17 (81.67-103.24)	0.076	102.02 (95.33-106.93)	0.043^*^
	High	8 (5)	94.66 (80.90-106.20)		84.96 (66.33-95.27)		93.87 (75.09-102.32)	

DM, Diabetes mellitus; CV, Cardiovascular, COPD, Chronic obstructive pulmonary disease, IQR, Inter quartile range,

* P < 0.05, by Mann Whitney U Test or Kruskal-Wallis Test

The logistic regression analysis indicates significant correlations between DM risk, CV risk, and lung function impairment shown in Table 3. Individuals with high to very high DM risk exhibit a significant likelihood of lung function impairment, with an odd ratio (OR) of 11.88 (95% CI at 1.41–100.0) and a significant adjusted odd ratio (aOR) of 9.06 (95% CI at 1.05–78.53, p < 0.05). Participants in the high to very high CV risk group showed a significantly increasing risk of lung function impairment, as indicated by an OR of 2.97 (95% CI at 1.12–7.90) and an aOR of 1.84 (95% CI at 0.60–5.69), although this significance was lost post-adjustment. High-risk individuals for COPD showed not statistically significant elevation in lung function impairment risk, with an OR of 4.24 (95% CI at 0.93–19.34) and an aOR of 3.50 (95% CI at 0.62–19.60).

**Table 3:** Logistic regression analysis of lung function and chronic health risk (N = 145).

Variable	Category	Lung function, n (%)			Lung function impairment	
Normal	Impaired	Missing	OR (95% CI)	#aOR (95% CI)
DM risk level	Low risk	33 (91)	1 (3)	2 (6)	Ref	Ref
	Low to moderate	26 (90)	3 (10)	–	3.81 (0.37–38.78)	3.72 (0.36–38.92)
	Moderate to high	41 (84)	7 (14)	1 (2)	5.63 (0.66–48.12)	4.15 (0.47–36.52)
	High to very high	25 (66)	9 (24)	4 (10)	11.88 (1.41–100.0)^*^	9.06 (1.05–78.53)^*^
CV risk level	Low to moderate	98 (87)	11 (10)	4 (3)	Ref	Ref
	High to very high	27 (69)	9 (23)	3 (8)	2.97 (1.12–7.90)^*^	1.84 (0.60–5.69)
COPD risk level	Low risk	120 (83)	17 (12)	7 (5)	Ref	Ref
	High risk	5 (62)	3 (38)	–	4.24 (0.93–19.34)	3.50 (0.62–19.60)

DM, Diabetes mellitus; CV, Cardiovascular, COPD, Chronic obstructive pulmonary disease; OR, odds ratio,

* P < 0.05, by Logistic regression analysis. **#** Adjust for sex, age, education, and income.

## Discussion

This study investigates associations between respiratory health and chronic disease risk factors among agricultural residents in Chiang Mai, Thailand. Our research uniquely examines this relationship within Northern Thailand’s agricultural population, who face distinct occupational exposures, environmental pollutants, and socioeconomic conditions that may affect lung function differently than urban dwellers. By analyzing chronic disease risk scores alongside spirometry results, we provide insights specific to this understudied population. The demographic characteristics of our sample and their potential relationship to respiratory outcomes offer preliminary understanding of health patterns in this community, though convenience sampling limits generalizability. This work contributes to understanding how environmental and occupational factors may influence respiratory health in rural settings, highlighting the need for region-specific health interventions and further research using randomized sampling methods.

The observed gender disparity in lung function, where men exhibit lower forced vital FVC and FEV1/FVC ratios compared to women, aligns with existing literature attributing these differences to physiological and occupational factors, such as heavier physical labor and increased exposure to occupational hazards among men. However, women demonstrate lower FEV1% predicted than men, indicating a complex interplay of factors influencing lung function across genders. Additionally, the observed relationship between age and lung function does not follow the expected pattern of age-related decline seen in previous studies. Individuals over 60 in our study exhibited lung function values that did not show a significant decline, suggesting that other factors, such as long-term adaptation to environmental exposures or selection bias in participant recruitment, may play a role. Further research is needed to explore these discrepancies [20–23]. Overall, these findings underscore the multifaceted nature of lung function disparities, necessitating a nuanced understanding of gender and age-related influences on respiratory health.

In this study, lower-income individuals exhibited unexpectedly favorable spirometry outcomes, particularly in FEV1/FVC% predicted ratios, which diverges from previous findings that associated lower socioeconomic status (SES) with poorer lung function outcomes. This discrepancy may be attributed to the physically demanding nature of agricultural work prevalent among lower-income populations, potentially enhancing lung function through increased physical activity. Additionally, these individuals may experience reduced exposure to air pollutants due to residing in less industrialized areas or engaging in different agricultural practices, which could further benefit lung health. Previous research has shown that higher household income is generally linked to lower risks of pulmonary diseases, such as COPD and asthma, indicating a complex relationship between income and lung function influenced by occupational, environmental, and lifestyle factors in rural settings [24,25]. Therefore, public health interventions should consider these unique contextual factors to effectively address lung health disparities across various income levels [26,27].

The findings from our studies indicate that individuals with lower DM risk exhibit significantly better lung function, as evidenced by higher FVC% predicted values. This suggests that chronic inflammation and reduced lung elasticity associated with DM adversely affect pulmonary health [28]. Similarly, patients at low risk for COPD demonstrate a notably higher FEV1/FVC ratio compared to those at high risk, highlighting the early onset of airflow limitations in COPD [29]. The presence of comorbidities, such as diabetes and metabolic syndrome, exacerbates respiratory dysfunction, emphasizing the need for integrated care approaches to manage these chronic conditions effectively [30,31]. Our studies collectively underscore the critical importance of managing DM and COPD to preserve lung function and enhance overall health outcomes, advocating for proactive monitoring and intervention strategies in at-risk populations [32].

The logistic regression analysis reinforced the strong association between high DM and CV risk levels and lung function impairment. Relied on several previous studies indicate that chronic inflammation due to DM can lead to significant lung function decline, particularly in individuals with poor glycemic control, as evidenced by a restrictive pattern of lung disease observed in diabetic patients with elevated inflammatory markers [28]. Furthermore, metabolic syndrome (MS) has been linked to increased severity of respiratory dysfunction, with studies showing that individuals with MS exhibit more pronounced lung function impairment compared to those without [29,33]. The presence of MS exacerbates the risk of cardiopulmonary morbidity and mortality, highlighting the critical need for integrated disease management strategies that address both respiratory and metabolic health [34]. This multifaceted approach is essential, as it can potentially mitigate the debilitating effects of these chronic conditions on lung function and overall health [35]. Thus, addressing both DM and CV risks is vital for improving respiratory outcomes in affected populations.

This research elucidates the considerable influence of demographic and health factors on pulmonary function in Chiang Mai’s agricultural residents. The observed associations between elevated chronic disease risks and diminished lung performance indicate a significant exacerbation of respiratory health challenges. The results emphasize the necessity for specialized public health initiatives to alleviate chronic disease risks and enhance lung function, with particular attention to older adults and economically disadvantaged groups. Subsequent initiatives should prioritize integrated approaches that address both respiratory and metabolic health to alleviate the compounded vulnerabilities of these at-risk populations.

## Supporting information

S1 File Data.. (XLSX)
